# Supplementary material for: Hybrid Models and Biological Model Reduction with PyDSTool
Source: PLoS Comput Biol. 2012 Aug 9;8(8):e1002628. doi: 10.1371/journal.pcbi.1002628 (PMC3415397; doi:10.1371/journal.pcbi.1002628)
Supplement: Text S4 — Complete source code for the PyDSTool package (version 0.88.120504). Includes API documentation and help files linking to web pages. This file is identical to the current public release on Sourceforge.net. (ZIP) [file pcbi.1002628.s004.zip › PyDSTool/html/PyDSTool.MProject.intModelInterface-class.html]

xml version="1.0" encoding="ascii"?


PyDSTool.MProject.intModelInterface


| Home | Trees | Indices | Help | PyDSTool |
| --- | --- | --- | --- | --- |

Package PyDSTool ::
Module MProject ::
Class intModelInterface

[hide private]

[frames] | no frames]

# Class intModelInterface

source code

```
 object --+        
          |        
dsInterface --+    
              |    
 ModelInterface --+
                  |
                 intModelInterface
```

---

Interface providing internal evaluation criteria between models.
Optional conditions (object) argument used to specify these criteria.


|  |  |
| --- | --- |
| Instance Methods | [hide private] |

|  |  |
| --- | --- |
| \_\_init\_\_(self, model, conditions=None, compatibleInterfaces=None, test\_traj=None)  Set model that generates test trajectories from which the dictionary of conditions can be imposed on a connected model. | source code |

|  |  |
| --- | --- |
| ensure\_has\_test\_traj(self)  Cause recomputation of test trajectory if not already present in model, returning boolean for whether recomputation was performed. | source code |

|  |  |
| --- | --- |
| has\_test\_traj(self) | source code |

|  |  |
| --- | --- |
| compute\_traj(self, need\_init=True, new\_args=None) | source code |

|  |  |
| --- | --- |
| get\_test\_traj(self, force=False)  Called by another interface. | source code |

|  |  |
| --- | --- |
| initialize\_model(self)  Return any unique model-specific settings here, as a dictionary with keys that can include initial conditions, parameters, tdata, algorithmic parameters. | source code |

**Inherited from `ModelInterface`**:
`AuxVars`,
`Jacobian`,
`JacobianP`,
`MassMatrix`,
`Rhs`,
`__call__`,
`evaluate`,
`get`,
`postprocess_test_traj`,
`prepare_conditions`,
`set`,
`setup_conditions`

**Inherited from `ModelInterface`** (private):
`_get_initiator_cache`

**Inherited from `dsInterface`**:
`query`

**Inherited from `object`**:
`__delattr__`,
`__getattribute__`,
`__hash__`,
`__new__`,
`__reduce__`,
`__reduce_ex__`,
`__repr__`,
`__setattr__`,
`__str__`


|  |  |
| --- | --- |
| Class Variables | [hide private] |

**Inherited from `ModelInterface`** (private):
`_trajname`

**Inherited from `dsInterface`** (private):
`_querykeys`,
`_setkeys`


|  |  |
| --- | --- |
| Properties | [hide private] |

**Inherited from `object`**:
`__class__`


|  |  |
| --- | --- |
| Method Details | [hide private] |

|  |  |
| --- | --- |
| \_\_init\_\_(self, model, conditions=None, compatibleInterfaces=None, test\_traj=None)  *(Constructor)* | source code |

```
Set model that generates test trajectories from which the dictionary
of conditions can be imposed on a connected model.

If no conditions are specified then the model is trivially wrapped in
  an "empty" interface.

Optionally, a dummy test traj can be supplied in case of a dummy interface
for a trivial condition test that does not need to evaluate a trajectory
to determine the result.
```

Overrides: object.\_\_init\_\_

|  |  |
| --- | --- |
| get\_test\_traj(self, force=False) | source code |

Called by another interface. Return model's test trajectory, using any
post-processing specified by user-defined process\_test\_traj method.

Use force option if model is known to have changed and trajectory
needs refreshing.

Overrides: dsInterface.get\_test\_traj

|  |  |
| --- | --- |
| initialize\_model(self) | source code |

Return any unique model-specific settings here, as a dictionary with
keys that can include initial conditions, parameters, tdata, algorithmic
parameters. Use the same keys that are suitable for a call to the
Model.set method, i.e. 'pars', 'ics', 'tdata', and 'algparams'.

Override in a sub-class to use. This method will be called before any
trajectory computation of the model.

  


| Home | Trees | Indices | Help | PyDSTool |
| --- | --- | --- | --- | --- |

|  |  |
| --- | --- |
| Generated by Epydoc 3.0.1 on Fri May 4 15:24:06 2012 | http://epydoc.sourceforge.net |
